# A New Nerve

**Published:** 1879-01

**Authors:** 


A New Nerve.—In a communication to the French Academy, Cyon claims that the eighth pair of cerebral nerves contain two nerves of entirely distinct senses—the auditory and the nerve of space ("Raumnerf"). He considers the latter the source of all our ideas of extension, and of the three dimensions of space.—*The Doctor.*

